# Study on metabolic differences and microbiota distribution in patients with infection-induced kidney stones by gender

**DOI:** 10.1186/s12866-026-05229-8

**Published:** 2026-06-08

**Authors:** Endi Zhang, Zhichao Chi, Xuming Zhang, Jianxing Li, Chaoyue Ji, Weiguo Hu

**Affiliations:** Department of Urology, Beijing Tsinghua Changgung Hospital, School of Clinical Medicine, Tsinghua Medicine, Tsinghua University, Beijing, 102218 China

**Keywords:** Infection stones, Microbiota, Metabolic assessment, Gender

## Abstract

**Objective:**

This study aims to investigate the distribution patterns of microbiota and stone composition in patients with infection-induced stones across different genders, and to elucidate the differences between stone composition and metabolism.

**Methods:**

Clinical data from 355 patients with infection stones (confirmed by postoperative stone composition analysis) who were hospitalized and treated at our center from January 2016 to December 2023 were retrospectively analyzed. The collected data included preoperative mid-stream urine culture results, serum biochemical parameters, 24-hour urinary metabolic indices, and postoperative stone composition analysis. The patients were divided into two groups based on gender to analyze the distribution of stone composition and microbiota. Metabolic differences between the groups were assessed based on blood biochemical markers and 24-hour urinary metabolic test results.

**Results:**

Of the 355 cases, struvite-carbonate apatite mixed stones were most frequent overall and in females (*P* < 0.05), while pure struvite stones dominated in males (*P* < 0.05). Microbiota distribution showed *E. coli* was most common in females and *P. aeruginosa* in males (*P* < 0.05). Females had significantly higher serum phosphorus, chlorine, and eGFR (*P* < 0.05), whereas males had higher blood urea nitrogen, creatinine, and uric acid (*P* < 0.05). Additionally, 24-hour urinary phosphorus, chlorine, and sodium excretions were greater in males (*P* < 0.05).

**Conclusion:**

This study found significant gender differences in the microbial distribution, stone composition, and metabolic characteristics of patients with infection stones. These findings suggest that more comprehensive metabolic evaluation and urine culture may be of help in optimizing clinical management. Additionally, the study observed that male patients with infection stones tended to have relatively poorer renal function-related indicators, suggesting that this population might benefit from more timely and effective clinical attention and intervention.

## Introduction

Urolithiasis is a common disease worldwide. Over the past few decades, although its incidence has fluctuated slightly, it has generally shown a continuous upward trend, placing a substantial burden on healthcare systems globally [1]. Data from the National Health and Nutrition Examination Survey (NHANES) indicate that the prevalence of urolithiasis in the United States has been increasing over recent decades, and it is projected to generate an additional economic burden of approximately $1.24 billion annually in the future [2]. In China, a cross-sectional study has revealed that approximately 1 in 17 adults is affected by this condition [3].

Infection stones are a distinct subtype of urolithiasis, accounting for approximately 5%-15% of all urinary stones [4, 5]. Their formation is closely associated with recurrent urinary tract infections caused by urease-producing bacteria. These stones are primarily composed of magnesium ammonium phosphate (struvite) and/or carbonate apatite and can rapidly develop into large staghorn calculi [6]. Previous studies have found significant gender differences in the prevalence of urolithiasis, with an overall higher incidence in men (approximately 19%) than in women (approximately 9%); however, this gap has been gradually narrowing in recent years [7]. Nevertheless, due to the higher incidence of urinary tract infections in women, the prevalence of infection stones is significantly higher in females than in males, with a female-to-male ratio of approximately 2:1 [8, 9]. Among the associated urinary tract infections, the most common non-urease-producing and urease-producing bacteria are *Escherichia coli* and *Proteus mirabilis*, respectively [10].

Urinary stones induced by bacterial infection often exhibit a complex composition and are not exclusively composed of typical infection-related components; they frequently present as mixed stones, suggesting that other factors may also participate in the lithogenic process. Studies have indicated that even patients with pure infection stones may have underlying metabolic disorders. Therefore, assessing metabolic factors in these patients could help optimize treatment and prevent stone recurrence [4]. To date, a substantial body of research has primarily focused on treatment strategies for urinary stones, while studies specifically addressing the metabolic characteristics of infection stones remain relatively limited, particularly those delving into gender-based differences. In light of this, the present study retrospectively analyzed data from 355 patients with infection stones who were hospitalized at our center from January 2016 to December 2023. From a gender perspective, we summarized the patients’ general characteristics, blood and urinary parameters, microbiota distribution, and stone composition features. This analysis aims to provide insights for developing individualized prevention strategies, enhancing therapeutic outcomes, and reducing the risk of recurrence.

## Materials and methods

### Study patients

This study was approved by the Ethics Committee of Beijing Tsinghua Changgung Hospital, affiliated with Tsinghua University. We retrospectively included 355 patients diagnosed with infection stones at our center between January 2016 and December 2023. All enrolled patients with infection stones were confirmed postoperatively by Fourier transform infrared spectroscopy for stone composition analysis, with infection stone components (including magnesium ammonium phosphate hexahydrate, carbonate apatite, and ammonium urate) accounting for ≥ 50% of the stone composition [11]. Stones were classified into pure infection stones (containing only one of the following: magnesium ammonium phosphate hexahydrate, carbonate apatite, or ammonium urate) and mixed infection stones (containing two or more infection components, or a mixture of infection components and non‑infection components such as calcium oxalate). For patients with multiple hospital visits, only the data from their first visit were collected for statistical analysis. All participants provided written informed consent and had complete baseline clinical data. Exclusion criteria were as follows: (1) severe organ failure (cardiac, pulmonary, hepatic, or renal); (2) congenital kidney diseases such as medullary sponge kidney or renal tubular acidosis; (3) neurogenic bladder; (4) congenital urinary tract malformations; (5) use of antibiotics, diuretics, or other medications that alter urinary flow rate within one week before admission; and (6) prior urological intervention within three months.

### Research methods

We collected and organized the patients’ baseline data, including age, body mass index (BMI), medical history (hypertension, diabetes), as well as laboratory findings (serum biochemistry, 24-hour urine analysis, urine culture, and stone composition analysis). The diagnostic criteria for comorbidities were as follows: the diagnosis of hypertension was based on the 2020 International Society of Hypertension International Hypertension Practice Guidelines [12]; the diagnosis of diabetes was based on the 2021 American Diabetes Association Classification and Diagnosis of Diabetes [13].

### Serum biochemistry and 24-hour urine sample collection

Blood samples were collected from all patients via venipuncture in the morning after an overnight fast. Routine biochemical tests included serum electrolytes, blood urea nitrogen, creatinine, uric acid, and parathyroid hormone (PTH), and the estimated glomerular filtration rate (eGFR) was calculated. The 24-hour urine collection began at 8:00 AM on the second day of admission and ended at 8:00 AM on the third day. Hydrochloric acid was added at the time of the initial collection to stabilize the urine components. Subsequently, urinary parameters, including urine pH, total volume, and excretion rates of calcium, phosphorus, sodium, potassium, chloride, and uric acid, were measured using a certified automated analyzer following standardized methods. During the urine collection period, patients were instructed to follow standardized guidance: avoid vigorous physical activity and keep a detailed record of their dietary intake.

### Midstream urine and random urine specimen collection

After admission and before initiating antibiotic therapy, a 10 mL midstream urine sample was collected from each patient using a sterile collection tube and immediately sent for bacterial culture. If immediate testing was not possible, the specimen was stored in a refrigerator at 4 °C and analyzed within 8 h. A positive urine culture was defined as a colony count ≥ 1 × 10^5 CFU/mL. All culture results were reported by the microbiology laboratory of our center in accordance with clinical laboratory operating procedures.

### Stone composition analysis

All enrolled patients with infection stones were confirmed postoperatively by Fourier transform infrared spectroscopy for stone composition analysis. Stones were classified into pure infection stones (containing only one of the following: magnesium ammonium phosphate hexahydrate, carbonate apatite, or ammonium urate) and mixed infection stones (containing two or more infection components, or a mixture of infection components and non‑infection components such as calcium oxalate), with infection stone components (including magnesium ammonium phosphate hexahydrate, carbonate apatite, and ammonium urate) accounting for ≥ 50% of the stone composition [11].

### Statistical analysis

SPSS software (version 27.0) was used for statistical analysis. Measurement data conforming to a normal distribution are presented as the mean ± standard deviation (SD), while count data are presented as frequency (N) and percentage (%). Comparisons between groups were performed using the chi-square test, t-test, and Fisher’s exact test. For measurement data not conforming to a normal distribution, results are expressed as median [M (P25ཞP75)], and comparisons between groups were performed using the Mann-Whitney U test. For all statistical analyses, a *P*-value < 0.05 was considered to indicate a statistically significant difference.

## Results

### Baseline characteristics of patients

Among the 355 patients with infectious stones, there were 120 males (33.8%) and 235 females (66.2%). The mean age was 50.88 years for males and 49.41 years for females, with no statistically significant difference between the two groups (*P* > 0.05). Furthermore, no significant statistical differences were observed between genders regarding comorbidities (hypertension, diabetes) and body mass index (BMI) (*P* > 0.05) (Table 1).

**Table 1:** Baseline Characteristics of Patients

Baseline Characteristics	Male	Female	$\:\boldsymbol{x}$^2^/t	*P*-value
N	120(33.8)	235(66.2)		
Age	50.88 ± 13.58	49.41 ± 12.32	1.030	0.304
Hypertension			1.666	0.197
Yes	34(9.6)	52(14.6)		
No	86(24.2)	183(51.5)		
Diabetes			0.005	0.943
Yes	11(3.1)	21(5.9)		
No	109(30.7)	214(60.3)		
BMI(kg/m^2^)	24.42 ± 3.82	24.60 ± 4.89	-0.345	0.730

### Distribution of stone components

Based on the stone composition, stones containing only magnesium ammonium phosphate hexahydrate (MAP), carbonate apatite (CA), or ammonium urate (AAU) were defined as pure infectious stones, while those containing two or more components were classified as mixed infectious stones. Among the 355 stone samples, MAP was the most common single-component stone (10.2%). Regarding mixed infectious stones, the combination of MAP and CA was the most frequently observed (50.1%), followed by the combination of CA and calcium oxalate monohydrate (COM) (Table 2).

**Table 2:** Stone Composition Analysis

Component	$\:\mathrm{N}$	%
Single		
MAP	36	10.2
CA	22	6.2
Mixed		
MAP + CA	178	50.1
CA + COM	46	13.0
CA + COD	3	0.8
MAP + CA+AAU	20	5.6
MAP + CA+COM	21	5.9
CA + COD+COM	29	8.2
Total	355	100

Comparison of stone components between genders showed that the proportion of pure MAP stones was significantly higher in male patients than in female patients (*P* < 0.05). Additionally, the proportions of mixed stones composed of CA + COM and those composed of CA + COM+calcium oxalate dihydrate (COD) were also higher in male patients compared to female patients, with the differences being statistically significant (*P* < 0.05). In contrast, the proportion of mixed MAP + CA stones was significantly higher in female patients than in male patients, and this difference was statistically significant (Table 3).

**Table 3:** Distribution Characteristics of Stone Components

Stone Components	Group(%)			$\:\boldsymbol{x}$^2^\Fisher	*P*-value
	Male	Female	Total		
MAP	34(9.6)	2(0.9)	36(10.2)	65.840	<0.001
CA	8(2.3)	14(6.0)	22(6.2)	0.069	0.793
MAP + CA	29(8.2)	149(63.4)	178(50.1)	48.920	< 0.001
CA + COM	23(6.5)	23(9.8)	46(13.0)	6.196	0.013
CA + COD	2(0.6)	1(0.4)	3(0.8)	1.460	0.227
MAP + CA+AAU	5(1.4)	15(6.4)	20(5.6)	0.734	0.392
MAP + CA+COM	4(1.1)	17(7.2)	21(5.9)	2.172	0.141
CA + COD+COM	15(4.2)	14(6.0)	29(8.2)	4.533	0.033
Total	120(33.80)	235(66.20)	355(100)	-	-

### Comparing metabolic profiles between male and female patients

Analysis of serum metabolic parameters revealed that serum phosphorus, chloride, and estimated glomerular filtration rate (eGFR) were significantly higher in female patients than in male patients (*P* < 0.05), whereas blood urea nitrogen (BUN), serum creatinine, and serum uric acid levels were significantly higher in male patients (*P* < 0.05). No statistically significant differences were observed between the two groups regarding serum calcium, magnesium, potassium, sodium, and parathyroid hormone (PTH) levels (*P* > 0.05). Random urine pH also showed no significant difference between genders. Further analysis of the 24-hour urine test results indicated that urinary sodium, phosphorus, and chloride excretions were significantly higher in male patients than in female patients (*P* < 0.05). However, there were no statistically significant differences between the two groups in terms of urinary calcium, potassium, uric acid, and total urine volume (*P* > 0.05) (Table 4).

**Table 4:** Distribution Characteristics of Metabolic Parameters

Metabolic Parameters	Group		*P*-value
	Male(*n* = 120)	Female (*n* = 235)	
Serum Biochemistry			
Serum Calcium(mmol/L)	2.23 ± 0.13	2.25 ± 0.13	0.233
Serum Magnesium(mmol/L)	0.87 ± 0.08	0.88 ± 0.08	0.235
Serum Phosphorus(mmol/L)	1.15 ± 0.21	1.24 ± 0.22	< 0.001
Serum Potassium(mmol/L)	4.06 ± 0.41	4.00 ± 0.38	0.124
Serum Chloride(mmol/L)	106.79 ± 3.62	107.61 ± 3.04	0.035
Serum Sodium(mmol/L)	141.68 ± 2.74	142.09 ± 2.29	0.141
BUN(mmol/L)	8.05 ± 5.08	6.14 ± 4.04	< 0.001
Serum Creatinine(umol/L)	138.61 ± 104.25	88.10 ± 72.34	< 0.001
eGFR(mL/min)	71.97 ± 35.15	85.01 ± 33.27	< 0.001
Serum Uric Acid(µmol/L)	395 ± 106.91	319.93 ± 92.13	< 0.001
PTH(ng/mL)	69.22 ± 66.74	60.88 ± 51.98	0.233
Random Urine			
Urine pH(urine)	6.60 ± 0.64	6.63 ± 0.58	0.612
24-hour Urine			
Urine Calcium(mmol/d)	2.27 ± 2.23	2.48 ± 1.98	0.369
Urine Sodium(mmol/d)	116.04 ± 70.45	100.22 ± 62.08	0.031
Urine Potassium(mmol/d)	21.50 ± 12.87	20.16 ± 11.90	0.328
Urine Phosphorus(mmol/d)	11.11 ± 7.69	9.41 ± 6.92	0.036
Urine Chloride(mmol/d)	96.12 ± 61.25	81.28 ± 50.24	0.023
Urine Uric Acid(µmol/d)	1953.59 ± 1195.75	1779.49 ± 1064.61	0.163
Urine Volume(L/d)	2.30 ± 0.99	2.25 ± 0.81	0.613

*eGFR* Glomerular Filtration Rate, *PTH* Parathyroid Hormone, *BUN *Urea Nitrogen

### Distribution characteristics of bacterial flora

Among the 355 included patients with infectious kidney stones, midstream urine cultures were positive in 302 cases (85.1%). The most frequently detected bacterium was *Escherichia coli* (89 cases, 29.5%), followed by *Proteus mirabilis* (70 cases, 23.2%). Among urease-producing bacteria, *Proteus mirabilis* was the most common (23.2%), followed by *Pseudomonas aeruginosa* (24 cases, 7.9%); among non-urease-producing bacteria, *Escherichia coli* was predominant (29.5%), followed by *Enterococcus faecalis* (13 cases, 4.3%). The distribution of bacterial flora differed significantly between male and female patients. *Escherichia coli* was detected more frequently in female patients (25.2% vs. 4.3%, *P* = 0.001), whereas *Pseudomonas aeruginosa* was more common in male patients (5.3% vs. 2.6%, *P* = 0.001) (Table 5).

**Table 5:** Distribution of Bacterial Flora

Bacterial Flora	Group(%)			$\:\boldsymbol{x}$^2^\Fisher	*P*-value
	Male	Female	Total		
Escherichia coli	13(4.3)	76(25.2)	89(29.5)	14.448	< 0.001
Proteus mirabilis	15(5.0)	55(18.2)	70(23.2)	3.279	0.070
Pseudomonas aeruginosa	16(5.3)	8(2.6)	24(7.9)	16.530	< 0.001
Ureaplasma	8(2.6)	15(5.0)	23(7.6)	0.256	0.613
Klebsiella pneumoniae	5(1.7)	8(2.6)	13(4.3)	0.448	0.503
Enterococcus faecalis	7(2.3)	6(2.0)	13(4.3)	3.629	0.057
Candida	5(1.7)	8(2.6)	13(4.3)	0.448	0.503
Enterococcus faecium	1(0.3)	5(1.7)	6(2.0)	0.527	0.468
Streptococcus agalactiae	4(1.3)	0(0.0)	4(1.3)	9.399	0.002
Acinetobacter baumannii	0(0.0)	4(1.3)	4(1.3)	1.748	0.186
*Escherichia coli* and *Ureaplasma*	0(0.0)	5(1.7)	5(1.7)	2.193	0.139
*Escherichia coli* and *Enterococcus*	1(0.3)	3(1.0)	4(1.3)	0.051	0.822
*Proteusmirabilis* and *Escherichia coli*	0(0.0)	2(0.7)	2(0.7)	0.868	0.351
*Mycoplasma hominis* and *Ureaplasma*	0(0.0)	2(0.7)	2(0.7)	0.868	0.351
Other	16(5.3)	14(4.6)	30(9.9)	8.516	0.004
Total	91(30.13)	211(69.87)	302(100)	-	-

*Others*: Corynebacterium、Staphylococcus epidermidis、Klebsiella oxytoca, Staphylococcus saprophyticus, Providencia rettgeri, Morganella morganii, Alcaligenes faecalis, Enterobacter cloacae, Coagulase-negative Staphylococcus, Corynebacterium striatum, Staphylococcus warneri, Streptococcus anginosus, Gardnerella vaginalis, Enterobacter cloacae

### Multivariable logistic regression analysis

Analysis of the microbial distribution characteristics revealed significant gender differences in the distribution of Escherichia coli and Pseudomonas aeruginosa. To further explore potential risk factors associated with E. coli infection, a multivariable regression analysis was performed for E. coli. Given that the small number of positive cases for P. aeruginosa might affect model stability, multivariable regression analysis was not conducted for this pathogen. After performing collinearity diagnostics on blood urea nitrogen, serum creatinine, eGFR, serum uric acid, urinary phosphorus, urinary sodium, and urinary chloride, we ultimately included gender, eGFR, serum uric acid, 24‑hour urinary phosphorus, and pure MAP stones in the multivariable logistic regression analysis. The results showed that the risk of E. coli detection was significantly higher in females than in males (*P* < 0.001, OR 0.290, 95% CI [0.139, 0.603]), and that E. coli detection decreased with increasing 24‑hour urinary phosphorus excretion (*P* = 0.009, OR 0.946, 95% CI [0.908, 0.986]). No independent associations with E. coli detection were observed for eGFR (*P* = 0.579), serum uric acid (*P* = 0.337), or pure MAP stones (*P* = 0.802) (Table 6).

**Table 6:** Multivariable logistic regression analysis

Parameter	Regression coefficient	Standard error	Wald	*P*-value	OR (95% CI)
Gender	-1.239	0.374	10.985	< 0.001	0.290(0.139,0.603)
eGFR	0.002	0.004	0.308	0.579	1.002(0.995,1.010)
Serum Uric Acid	-0.002	0.002	0.923	0.337	0.998(0.995,1.002)
Urine Phosphorus	-0.055	0.021	6.875	0.009	0.946(0.908,0.986)
MAP	-0.145	0.578	0.063	0.802	0.865(0.279,2.685)

## Discussion

Urolithiasis is a common disease of the urinary system, characterized by high incidence and imposing a substantial economic burden on society. Its formation is influenced by multiple factors [14]. As an important type of urolithiasis, previous studies have generally believed that the formation of infectious stones is closely related to urinary tract infections and that they are more common in female patients. Without timely and effective diagnosis and treatment, patients with infectious stones may develop chronic renal failure or sepsis, which seriously threatens their life. Furthermore, residual stones after surgery can easily lead to recurrence [15]. Currently, related research primarily focuses on urinary tract infections themselves and their treatment, with relatively insufficient assessment of the metabolic characteristics of patients with infectious stones, particularly a lack of systematic analysis of metabolic differences between patients of different genders. To supplement research in this area, this study retrospectively collected and analyzed baseline data, blood and urine laboratory parameters, urine culture results, and stone composition data from 355 patients with infectious stones. Our analysis revealed significant differences in blood and urine biochemistry, bacterial flora distribution, and stone composition between male and female patients with infectious stones.

The present study found that mixed infection stones were the most common type among infection stones, while pure magnesium ammonium phosphate hexahydrate (MAP) stones and mixed infection stones containing calcium oxalate were more prevalent in male patients. Analysis of blood and urine parameters suggested that renal function impairment might be more severe in male patients with infection stones, and that their 24‑hour urinary excretion concentrations of sodium, phosphorus, and chloride were also higher. Human metabolism is influenced by multiple factors, including environment, diet, and medications, among which the kidneys play a key role. As the central organ regulating phosphorus metabolism, the kidneys primarily maintain phosphorus balance by modulating phosphate reabsorption; when renal function is impaired, phosphate reabsorption may be markedly inhibited, potentially leading to increased urinary phosphorus excretion and consequently higher phosphate ion concentrations in the urine [16]. Furthermore, several ion co‑transporters exist in the kidneys, such as the Na⁺–K⁺–Cl⁻ co‑transporter 2, which play important roles in maintaining the balance of sodium, chloride, calcium, and magnesium ions. Impairment of this co‑transporter could result in increased urinary excretion of sodium and chloride, accompanied by higher excretion of calcium and magnesium [17]. Theoretically, renal function impairment might lead to reduced phosphate reabsorption and increased urinary phosphorus excretion, thereby providing substrates for the formation of magnesium ammonium phosphate stones. However, given the cross‑sectional retrospective design of this study, a causal relationship could not be established. Future studies using dynamic measurements of glomerular filtration function and investigations of phosphate transporter expression are needed for verification. It is known that the formation of infection stones generally follows a pathological process involving urinary supersaturation, crystal nucleation, crystal growth, aggregation, and adhesion [18]. The above‑mentioned disturbances in urinary ion metabolism may create conditions conducive to crystal formation and stone growth.

Previous studies have suggested that the formation of calcium oxalate stones is closely associated with factors such as electrolyte imbalances, hyperoxaluria, or hyperuricemia in patients [19]. However, the findings of the present study did not reveal significant gender differences in calcium metabolism indicators, which may imply that calcium metabolism might not be the decisive driver of calcium oxalate stone formation. A prospective study has also indicated that hypercalcemia or hypercalciuria may not be key determinants of calcium oxalate stone formation; rather, urinary oxalate levels might be more relevant to stone formation, as calcium oxalate saturation in urine can increase rapidly even with a modest rise in oxalate concentration [20].

The excretion of urate is influenced by lifestyle and dietary habits, with high‑salt and high‑animal‑protein diets tending to promote urate excretion, and male patients often having a greater preference for such dietary patterns [11, 21, 22]. Moreover, some studies have suggested that androgens may further increase urinary oxalate excretion [23]. Due to limitations of the available data, the present study was unable to obtain urinary oxalate metabolic profiles for in‑depth analysis, which represents a limitation of this study. Future prospective studies incorporating a more comprehensive set of metabolic parameters are needed to further analyze and validate the above findings.

Stratified analysis by gender revealed significant differences in microbial distribution, with *Escherichia coli* being significantly enriched in female stone patients, while *Pseudomonas aeruginosa* was more common in male patients. The formation of infection stones is usually associated with infections caused by urease-producing bacteria, such as *Proteus mirabilis* and *Pseudomonas aeruginosa*. The urease produced by these bacteria hydrolyzes urea into ammonia and carbon dioxide; ammonia further reacts with water to form ammonium ions and hydroxide ions, thereby creating an alkaline urinary environment that promotes stone formation [15]. The present study found that pure magnesium ammonium phosphate hexahydrate (MAP) stones were more common in male patients. Previous studies have indicated that *Pseudomonas aeruginosa* possesses high urease activity, and its urease may significantly promote the formation of magnesium ammonium phosphate stones [24].

Escherichia coli was the most common bacterium in female patients, and the multivariable analysis also indicated that female patients might have a higher risk of E. coli infection. These findings are consistent with previous studies, which have reported a higher detection rate of non‑urease‑producing bacteria [25]. This could be attributable to the anatomical characteristics of females, which may predispose them to retrograde infection by intestinal flora. E. coli is a typical representative of non‑urease‑producing bacteria. Recent studies have suggested that the role of non‑urease‑producing bacteria in the formation of infection stones may need to be reconsidered, and that infection stone formation might not be mediated solely by classical urease‑producing bacteria, but may also involve synergistic interactions among microbial communities. Some recent studies have indicated that certain strains of E. coli may also possess urease‑producing capacity, and the proportion of urease‑positive strains might be increasing [26]. Furthermore, urease genes could potentially be transferred via plasmids. Since urease genes are located on plasmids, they might be transferred between bacterial species through transformation or conjugation; E. coli might consequently acquire urease‑producing ability [27]. This possibility, however, would require further validation through molecular experiments. Another possibility is that mixed infection by multiple microorganisms and polymicrobial biofilms might play a role. First, E. coli multiplies rapidly; in conventional culture, especially under alkaline conditions, its growth rate may exceed that of slower‑growing urease‑producing bacteria, which could lead to non‑detection of the latter. Second, urease‑producing and non‑urease‑producing bacteria may coexist within biofilms, achieving metabolic synergy and creating a localized alkaline microenvironment that could promote crystal formation even when urease activity is limited [28].

Multivariable analysis in this study suggested that as urinary phosphorus excretion increased, the risk of Escherichia coli infection might decrease, i.e., high urinary phosphorus could potentially inhibit E. coli colonization. High urinary phosphorus is often associated with a high‑protein or high‑salt diet, which might affect E. coli colonization by increasing urine output, or possibly by diluting the concentration of bacteria in the urine. In addition, phosphate has a buffering capacity, which might inhibit E. coli growth by altering the alkaline environment of urine. Nevertheless, this potential protective factor in female patients would need to be verified through further experiments.

It should be noted that midstream urine culture results may be influenced by bacterial load, as this method can only detect the dominant bacteria, and low‑abundance urease‑producing bacteria might not be detected in cases of polymicrobial infection. Furthermore, the microbial distribution profiles obtained from bacterial sequencing and stone culture could differ considerably, and since the present study did not perform both methods, this might have introduced some bias into the microbial detection results, which could be considered a limitation of this study. Nevertheless, given clinical practicability and cost‑effectiveness, midstream urine culture may still be the preferred method for diagnosing urinary tract infections in primary hospitals in China [11].

The observed gender differences in the formation of infection stones may be attributable to a complex interplay between inherent sex-specific metabolic disturbances and the colonization and activity of particular pathogenic microorganisms. This study found overlapping variations in metabolic parameters and microbial distribution between genders, suggesting that the metabolic background might interact with bacterial colonization and pathogenicity. For example, bacterial infection may alter urinary pH and ionic saturation, while metabolic abnormalities could also affect the local microenvironment. Such a “microbiota-metabolism” interaction might be involved in the development and progression of stones. The findings of the multivariable analysis in this study may indirectly support this possibility. Future research using longitudinal designs combined with multi-omics approaches could help further elucidate the mechanisms underlying this “microbiota-metabolism” interaction, which might provide a more solid basis for developing sex-specific strategies for the prevention, diagnosis, and treatment of infection stones.

This study is a single-center retrospective analysis, and selection bias may be present; therefore, the findings may not fully reflect stone formation patterns in different regions or ethnic populations. In addition, because data collection is challenging in retrospective studies, we were unable to account for medication use for chronic diseases or daily dietary habits in these patients, which could potentially influence stone composition, metabolic abnormalities, and microbial distribution. Indicators such as serum creatinine and eGFR may be affected by baseline physiological differences (e.g., muscle mass) and may not completely represent pathological injury. However, due to the nature of the study design, we were unable to collect additional data for background analysis, and our assessment of renal function relied on differences in serum creatinine and eGFR; consequently, the conclusions might be affected, and this is a limitation of the study. These limitations could have an impact on our findings. Future prospective cohort studies and multicenter studies using standardized metabolic testing methods and collecting more comprehensive clinical data are needed to further validate and extend the conclusions of this study. Nevertheless, the patients in our center came from various regions across mainland China, and our findings might still, to some extent, reflect the development, progression, and microbial distribution of infection stones in mainland China. Thus, they may still provide useful reference information for understanding gender differences in infection stones and for developing individualized prevention strategies.

This retrospective study observed gender-related differences in metabolism, microbiota, and stone composition among patients with infection stones. These findings suggest that gender may interact with metabolic and microbial factors in the process of infection stone formation. Male patients with infection stones showed serum renal function parameters (serum creatinine, eGFR), serum uric acid, and 24-hour urinary metabolic profiles that might indicate more pronounced metabolic abnormalities in this group, possibly related to low-phosphorus diet and adequate fluid intake. For female patients, preoperative midstream urine culture might be considered as a routine examination, with particular attention to *Escherichia coli*, and sensitive antibiotics could be selected based on antimicrobial susceptibility testing. Postoperative regular follow-up with urinalysis and urine culture might help prevent recurrence. The above observations might provide hypotheses for future prospective studies: for example, whether male patients with infection stones could benefit from more detailed renal function assessment and metabolic management (low-phosphorus diet, adequate fluid intake), and whether female patients might need to focus on *E. coli* in midstream urine culture. Furthermore, stone composition analysis showed considerable heterogeneity among all patients postoperatively, suggesting that it might help in formulating individualized follow-up strategies. However, all of the above associations would need to be further validated in prospective cohort or interventional studies.

## Data Availability

The datasets used and analyzed during the current study are available from the corresponding author on reasonable request.

## Abbreviations

eGFR: estimated glomerular filtration rate
BUN: blood urea nitrogen
PTH: parathyroid hormone
BMI: body mass index
MAP: magnesium ammonium phosphate hexahydrate
CA: carbonate apatite
COM: calcium oxalate monohydrate
COD: calcium oxalate dihydrate
AAU: ammonium acid urate
